# Supplementary material for: Loops, topologically associating domains, compartments, and territories are elastic and robust to dramatic nuclear volume swelling
Source: Sci Rep. 2022 Mar 18;12:4721. doi: 10.1038/s41598-022-08602-5 (PMC8933507; doi:10.1038/s41598-022-08602-5)
Supplement: Supplementary file 1 — Supplementary Information 1. [file 41598_2022_8602_MOESM1_ESM.pdf]

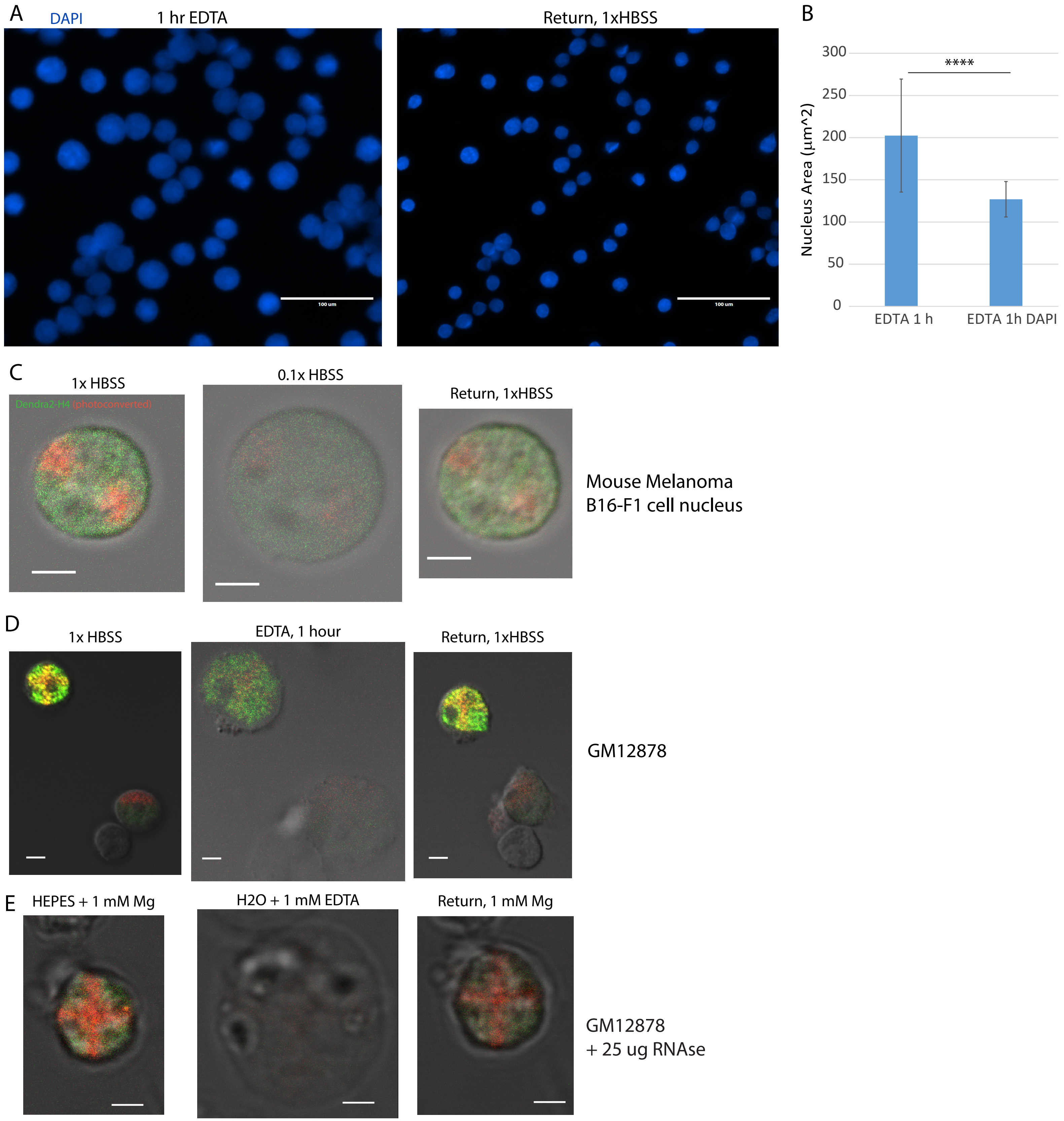

Supplementary Figure 1. A) DAPI staining after 1 h incubation of GM12878 nuclei in HEPES + 1mM EDTA (right) and return to 1x HBSS (right) shows chromatin fills expanded nucleus space (scale = 100 µm). B) The addition of DAPI after expansion reduces the size of the nuclei (EDTA 1h N = 115; DAPI N = 28; error bars = std. dev., **** $p<10^{-10}$, unpaired two-tailed t-test). C) Photoconverted pattern is also preserved upon a different approach to low salt expansion (dilution to 0.1x HBSS) and in a different cell type (mouse melanoma; scale = 3 µm). D) Photoconverted pattern in GM12878 cell is preserved after 1 hour of expansion, though the nucleus rotated ~90 degrees around the coverslip attachment point (see altered nucleolus position). (scale = 3 µm) E) RNase treatment does not affect nucleus expan-sion or fidelity of chromatin positioning after expansion. Nuclei were treated with 25 µg of RNAse (in 200 uL) for 10 min and then subjected to expansion. Scale bar = 2 µm.

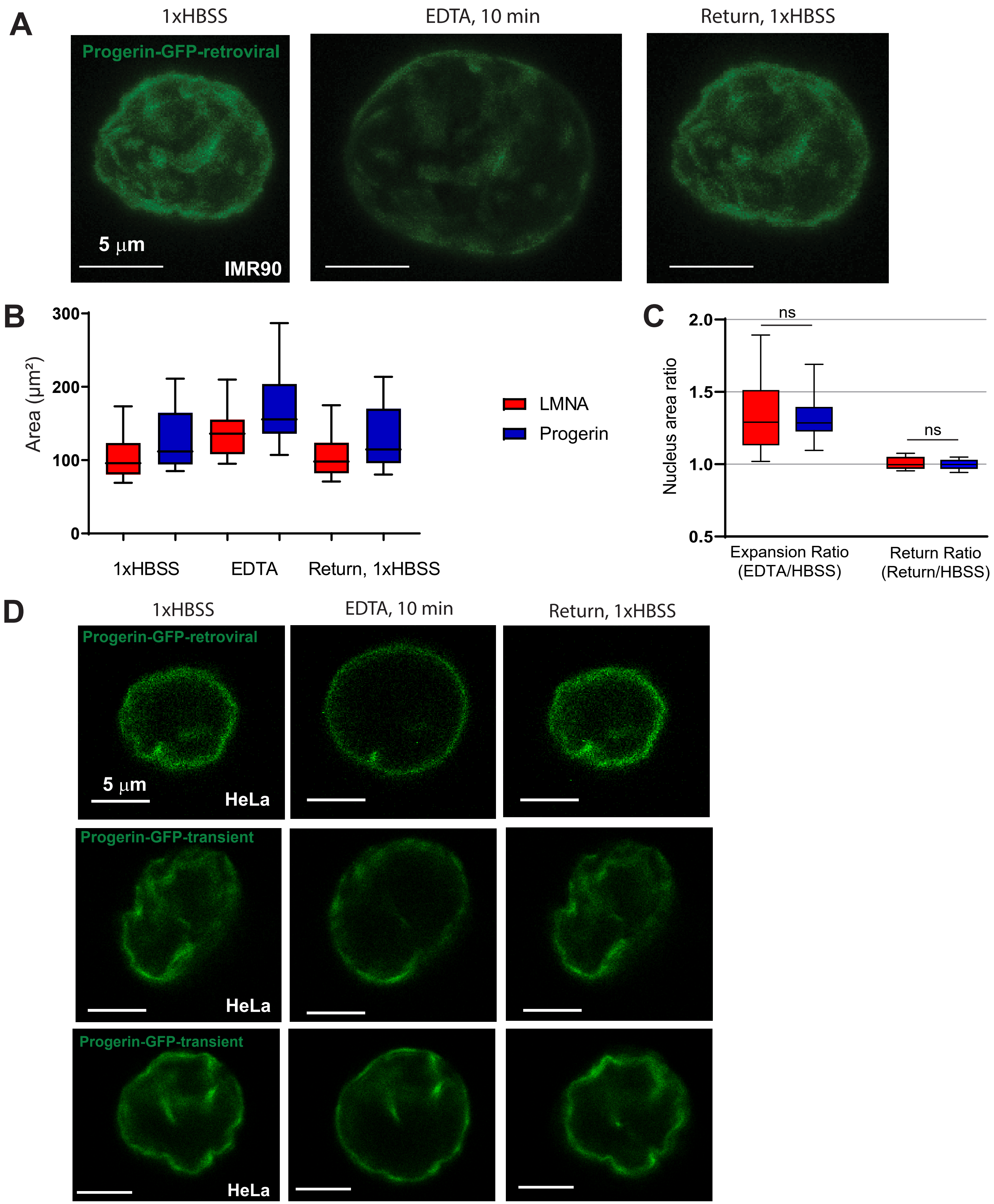

Supplementary Figure 2. A) IMR90 nuclei stably transfected with Progerin-GFP show wrinkles typical of progeria, but are able to expand and re-contract. Maximum projection of confocal Z-stack. B) IMR90 nuclei stably transfected with either WT LMNA-GFP (red, N=12) or Progerin-GFP (blue, N = 19) were quantified during expansion. Boxplots indicate 25th, median, and 75th percentiles. C) Comparing the expansion area ratio (EDTA/original 1xHBSS) and return area ratio (return / original 1xHBSS) shows no significant difference in relative expansion and re-contraction between LMNA and progerin transfected cells (ns: $p>0.45$, t-test). D) HeLa nuclei either transiently or stably transfected with progerin-GFP show smoothing of wrinkles with EDTA expansion and a return to original size in return to 1xHBSS. Confocal image central slice. All scalebars 5 microns.

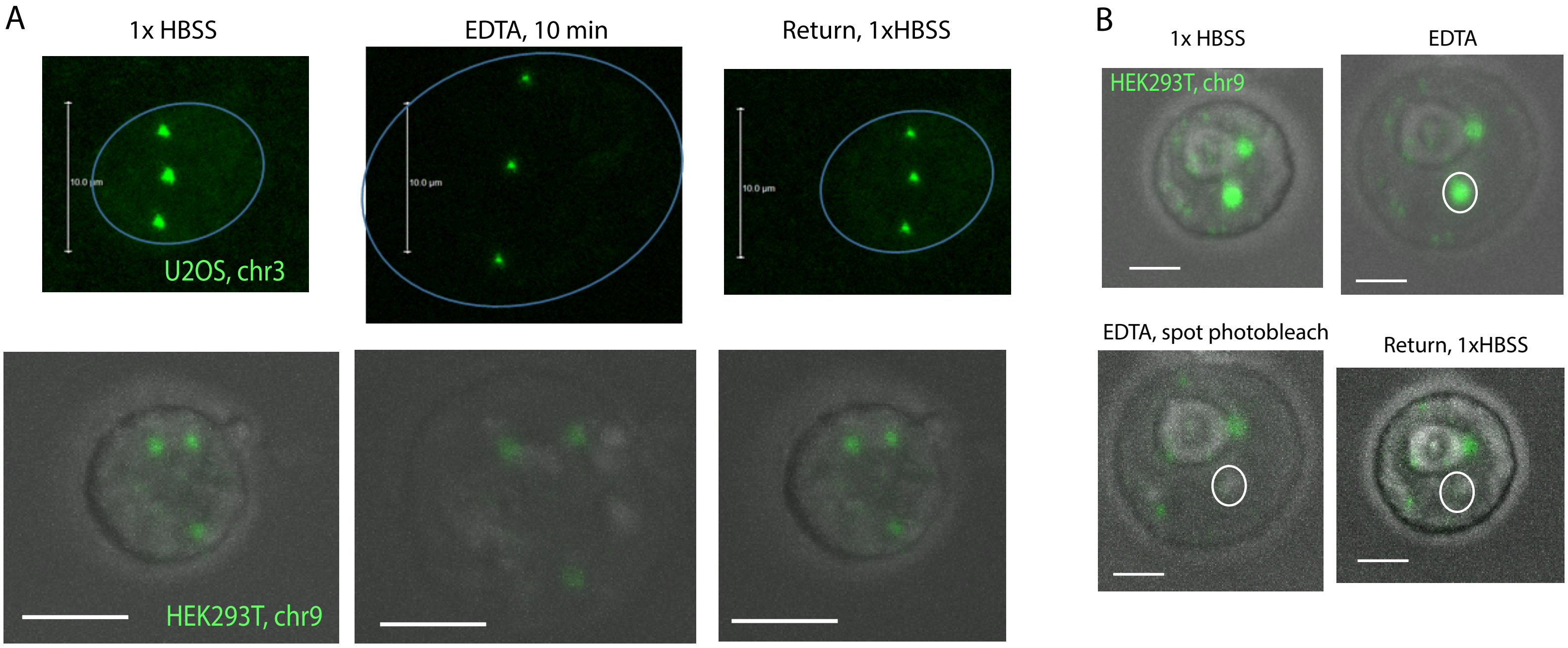

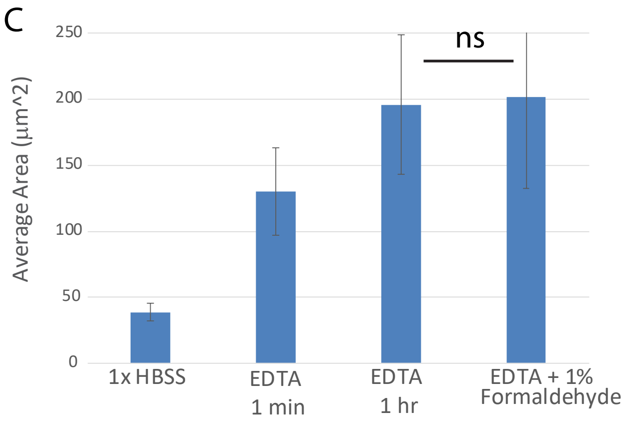

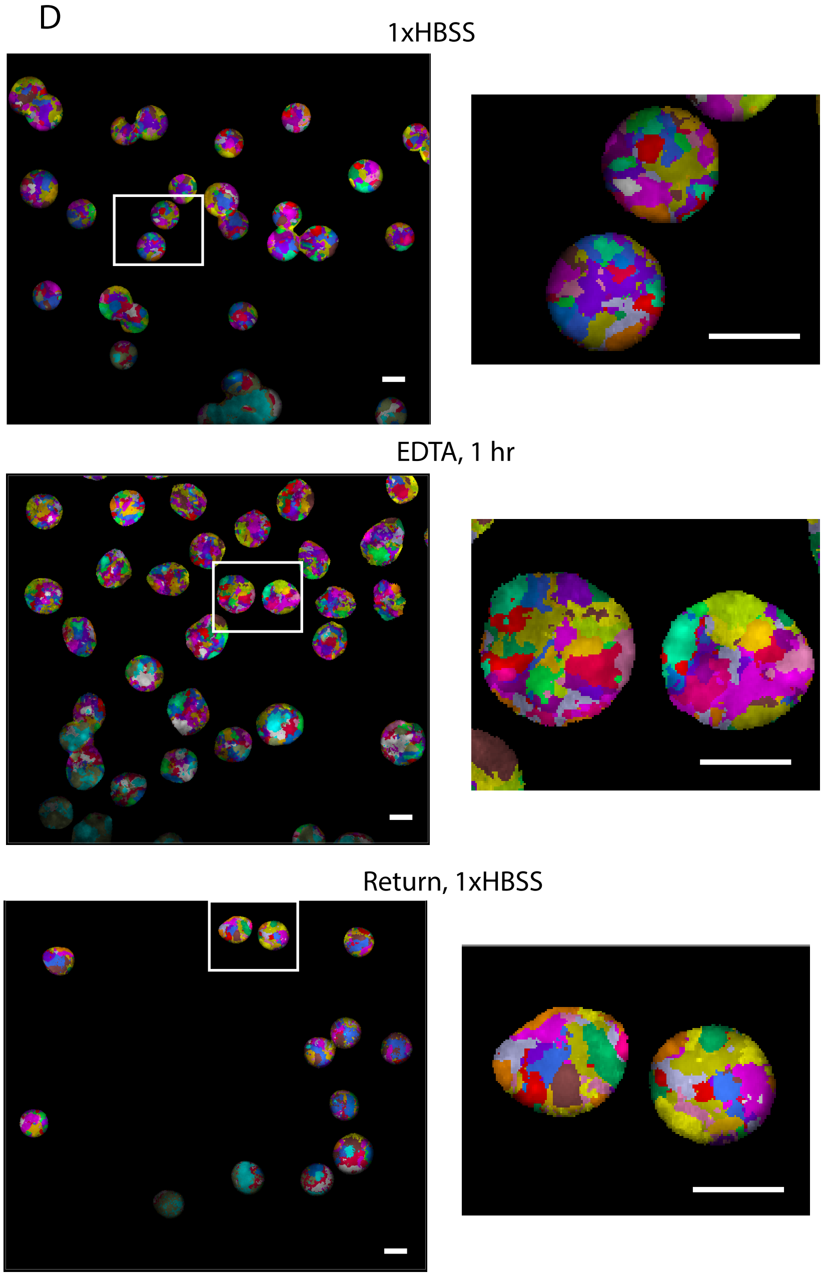

Supplementary Figure 3. A) U2OS cells transfected with Cas9-GFP targeting chromosome 3 pericentromeric region (top) and HEK293T cells transfected with Cas9-GFP targeting chromosome 9 pericentromeric region (bottom). Scale: top = 10 microns, bottom = 5 microns B) No fluorescence recovery is observed after return to 1xHBSS when a Cas9-GFP spot is photobleached (white circle) in the expanded state. (scalebar = 3 microns) C) Expanded nucleus size is preserved after crosslinking with 1% formaldehyde. (Bars show mean, error bars = std. dev. N = 83, 71, 116, 28). NS: p =0.67 by t-test. D) Multicolor FISH probes indicate the persistence of chromosome territories after expansion and contraction. Right panels = 4x zoom into the region in the white square in each left panel. Scalebars indicate the same distance (5 microns) in all panels.

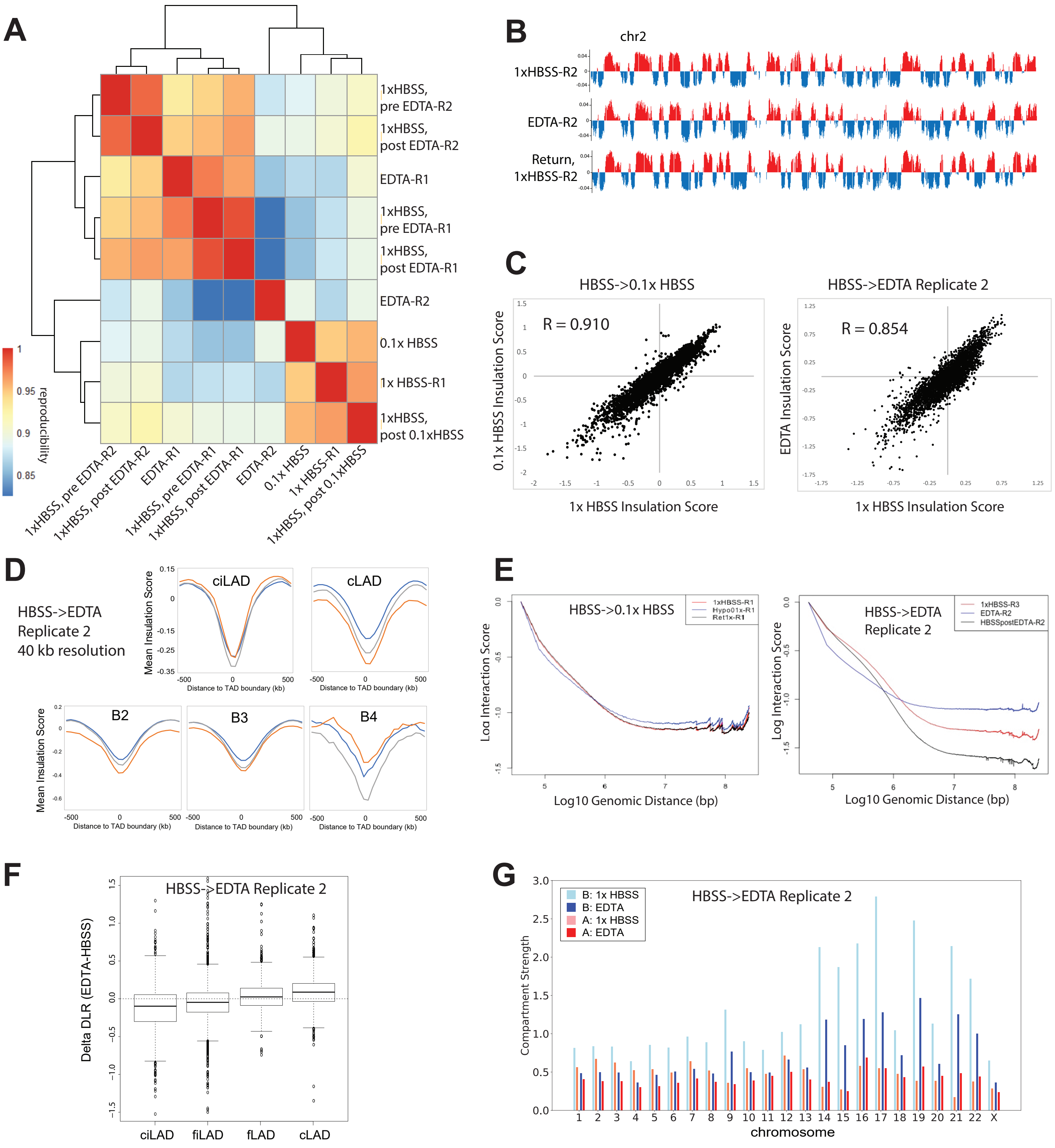

Supplementary Figure 4. A) Genome wide Hi-C contact corelation at 1 Mb resolution between all datasets. Spearman correlation-based reproducibility is calculated as described in the Methods. Replicates have some systematic differences, but expanded conditions (EDTA and 0.1xHBSS) are the more separate from non expanded condtitions in each replicate set. B) Compartment identity is conserved during EDTA expansion in replicate 2. chr2 eigenvector 1 is shown at 250 kb resolution. C) TAD insulation scores are preserved in expanded nuclei in both 0.1xHBSS and EDTA treatment replicate. Insulation scores shown for chr2 calculated with 520 kb insulation square size. D) Insulation score shifts around TAD boundaries in selected subcompartments and LAD types genome wide for EDTA replicate 2. 40kb resolution, 520 kb insulation square. 1xHBSS = blue, EDTA = orange, Return = grey. E) Log10 interactions vs. Log10 of genomic distance in base pairs for all 40 kb binned intrachromosomal interactions genome wide in the 0.1xHBSS expansion condition and the second EDTA replicate. A faster drop in local interactions and more long range interactions is observed in expansion. F) Changes in Distal Local Ratio for the whole genome separated into LAD status as classified by Kind et al. As in Figure 5D, but using Hi-C data from EDTA replicate 2. G) Compartment strength compared between 1x HBSS and EDTA for replicate 2.

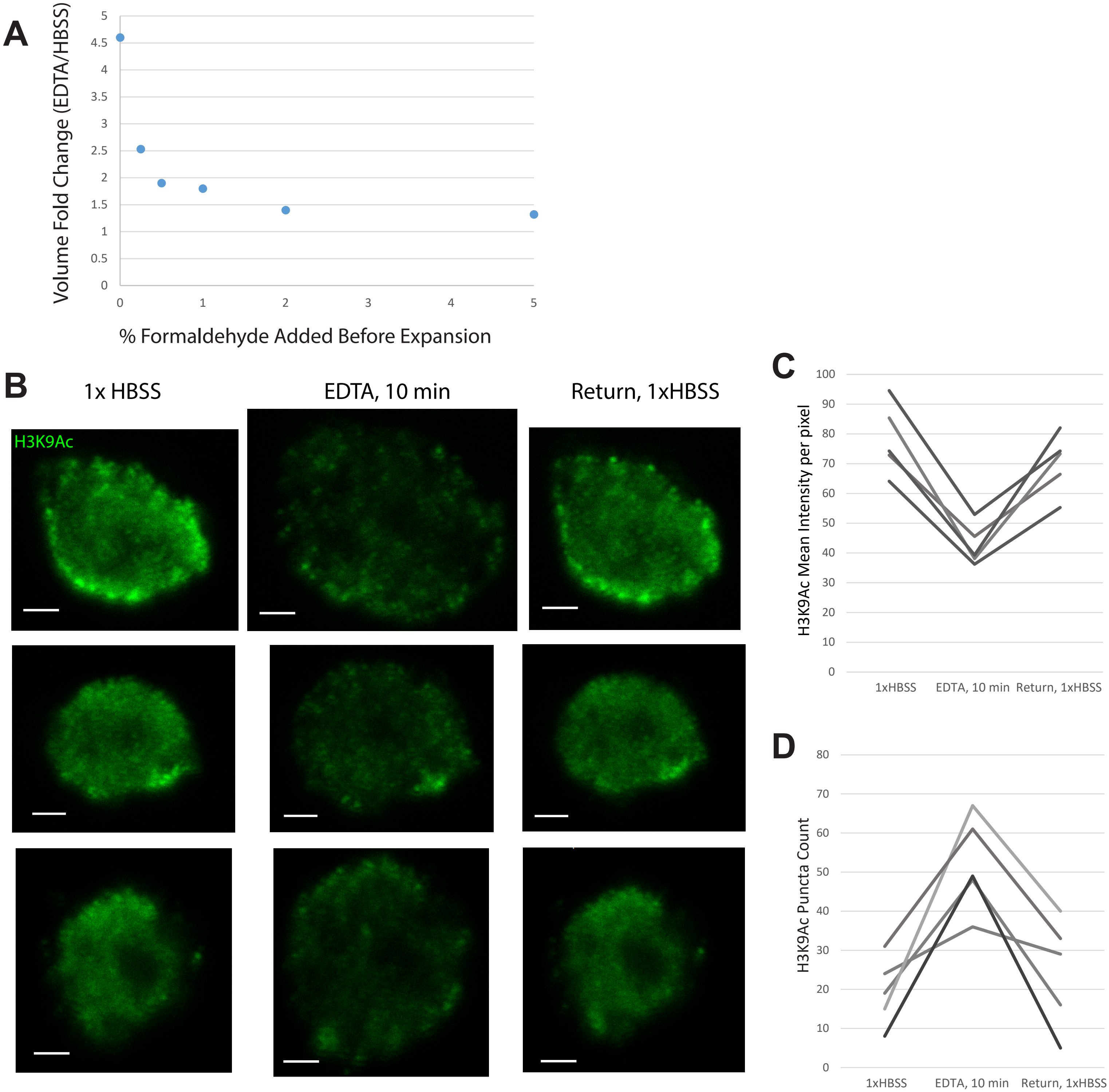

Supplementary Figure 5 A) Nuclei were crosslinked with increasing percentages of formaldehyde for 10 min prior to expansion with EDTA and the average fold change in nucleus volume after EDTA for 10 minutes is indicated. B) H3K9Ac immunostained (green) isolated GM12878 nuclei show more evident individual foci of this histone modification localization in the EDTA expanded state. After return to 1xHBSS, the original pattern is largely recapitulated. Images show a single confocal Z slice. Scale bars = 2 µm. C) The mean H3K9Ac intensity across each whole nucleus decreases during expansion. Lines connect the same nucleus. D) The number of detectable H3K27Ac puncta increases in the expanded state, as measured by thresholding the top 10% intensity and then detecting particles in ImageJ. Lines connect the same nucleus.

Supplementary Table 1. Hi-C Mapping Statistics

Condition	Replicate	Raw # Reads	# Both Sides Mapped	Valid Pairs	Unique Valid Pairs	% Cis	% Dangling Ends
1x HBSS	R1	228,519,373	158,543,659	133,626,690	127,083,536	38.0	10.6
0.1x HBSS, 1 h	R1	226,837,794	162,358,348	117,359,572	88,661,306	44.3	22.4
Return, 1xHBSS	R1	235,448,026	162,146,341	137,471,985	132,619,644	42.4	10.0
1x HBSS	R2	863,126,588	610,087,746	500,187,621	464,002,407	82.5	17.2
HEPES + 1mM EDTA, 1 h	R1	715,788,323	505,049,377	370,779,196	333,864,494	74.8	25.9
Return, 1xHBSS after EDTA	R1	706,960,951	484,478,770	389,402,191	350,509,296	76.4	19.1
1x HBSS	R3	128,374,188	100,671,274	66,707,754	63,677,166	68.8	33.4
HEPES + 1mM EDTA, 1 h	R2	116,329,893	85,695,816	47,499,071	43,718,226	42.1	42.7
Return, 1xHBSS after EDTA	R2	162,888,591	123,015,944	95,068,131	90,774,223	68.7	21.9

Supplementary Table 2. Factors enriched at GM12878 Loop Anchors All Rao Loops

Factor	# Loops with Factor	# Random with Factor	Total # Loops	% Loops with Factor	Log2(Real/ Random)
Sydh_Gm12878_Znf143	3633	465.53	7162	50.73	2.96
Sydh_Gm12878_Rad21	5239	737.13	7162	73.15	2.83
Sydh_Gm12878_Smc3	5263	813.33	7162	73.49	2.69
Uw_Gm12878_Ctcf	5324	857.25	7162	74.34	2.63
Haib_Gm12878_Rad21	5301	889.1	7162	74.02	2.58
Sydh_Gm12878_Ctcf	5428	1111.59	7162	75.79	2.29
Broad_Gm12878_Ctcf	5399	1129.56	7162	75.38	2.26
Uta_Gm12878_Ctcf	5370	1192.24	7162	74.98	2.17
Haib_Gm12878_Yy1	3240	746.7	7162	45.24	2.12

Rao Loops annotated as non-CTCF

Factor	# Loops with Factor	# Random with Factor	Total # Loops	% Loops with Factor	Log2(Real/ Random)	Log2(Enrichment in non-CTCF/CTCF)
Sydh_Gm12878_Corest	108	21.77	2882	3.75	2.31	1.08
Haib_Gm12878_Rxra	131	28.24	2882	4.55	2.21	0.23
Haib_Gm12878_Bclaf	420	91.87	2882	14.57	2.19	0.51
Sydh_Gm12878_Stat3	441	99.42	2882	15.30	2.15	0.97
Sydh_Gm12878_Znf143	1021	232.27	2882	35.43	2.14	-0.83
Sydh_Gm12878_P300b	391	92.15	2882	13.57	2.09	1.68
Sydh_Gm12878_Smc3	1418	338.12	2882	49.20	2.07	-0.63
Haib_Gm12878_Stat5a	465	114.86	2882	16.13	2.02	1.19
Sydh_Gm12878_Rad21	1410	353.38	2882	48.92	2.00	-0.83
Uta_Gm12878_Ctcf	1474	453.21	2882	51.15	1.70	
Broad_Gm12878_Ctcf	1448	457.57	2882	50.24	1.66	
Uw_Gm12878_Ctcf	1347	432.45	2882	46.74	1.64	
Sydh_Gm12878_Ctcf	1544	497.09	2882	53.57	1.64	

Rao Loops annotated as non-CTCF and with no ENCODE CTCF peaks

Factor	# Loops with Factor	# Random with Factor	Total # Loops	% Loops with Factor	Log2(Real/ Random)	Log2(Enrichment in non-CTCF/CTCF)
Sydh_Gm12878_P300b	58	9.42	372	15.59	2.62	2.22
Haib_Gm12878_P300	47	7.73	372	12.63	2.60	1.92
Sydh_Gm12878_Cfos	14	2.31	372	3.76	2.60	1.88
Sydh_Gm12878_Jund	25	4.29	372	6.72	2.54	2.14
Haib_Gm12878_Cebpb	45	8.61	372	12.10	2.39	1.59
Sydh_Gm12878_Chd1	41	7.89	372	11.02	2.38	1.57
Sydh_Gm12878_Tbp	83	16.98	372	22.31	2.29	1.55
Haib_Gm12878_Stat5a	51	10.92	372	13.71	2.22	1.39
Haib_Gm12878_Mta3	72	15.99	372	19.35	2.17	1.51
Haib_Gm12878_Pax5	89	20.46	372	23.92	2.12	1.35
Haib_Gm12878_Sp1	85	19.84	372	22.85	2.10	1.33
Haib_Gm12878_Taf1	65	15.35	372	17.47	2.08	1.37
Sydh_Gm12878_Ikzf1	47	11.86	372	12.63	1.99	1.57

Supplementary Table 3. Datasets used in Ridge and Random Forest Regression Models . All datasets are listed with their accession numbers in Encode or 4D Nucleome databases.

Dataset	Accession Number (FileType)
Repli-seq G1b	ENCFF001GNK (bigWig)
Repli-seq G2	ENCFF001GNN (bigWig)
Repli-seq S1	ENCFF001GNR (bigWig)
Repli-seq S2	ENCFF001GNT (bigWig)
Repli-seq S3	ENCFF001GNX (bigWig)
Repli-seq S4	ENCFF001GOA (bigWig)
MNase-seq	ENCFF000VME (bigWig)
FAIRE-seq	ENCFF000THZ (bigWig)
TSA-seq SON (K562)	4DNFIJC5NB7V (fastq)
TSA-seq Lamin AC (K562)	4DNFI7A2TXMD (fastq)
TSA-seq Lamin B (K562)	4DNFI9CPTQRL (fastq)
H2AFZ	ENCFF885XEM (bigWig)
H3K27Ac	ENCFF180LKW (bigWig)
H3K27Me3	ENCFF167NBF (bigWig)
H3K36Me3	ENCFF335VEM (bigWig)
H3K4Me1	ENCFF682WPF (bigWig)
H3K4Me2	ENCFF828CQV (bigWig)
H3K4Me3	ENCFF818GNV (bigWig)
H3K79Me2	ENCFF396JIR (bigWig)
H3K9Ac	ENCFF465KNK (bigWig)
H3K9Me3	ENCFF776OVW (bigWig)
H4K20Me1	ENCFF831WYD (bigWig)

Supplementary Movie 1. This movie shows an H3K9Ac stained isolated GM12878 nucleus as in Supplementary Figure 5B. Frames are taken every 10 seconds. At the beginning of the timelapse, the nucleus is in 1x HBSS. Then, two washes of EDTA in 1 mM HEPES are added and the nucleus expands rapidly.
